# The Role of the Caspian, Aral and Balkhash Lakes in the Spread and Preservation of Yersinia pestis in Eastern Europe and Central Asia in the 20th and 21st Centuries

**DOI:** 10.3390/pathogens15060568

**Published:** 2026-05-25

**Authors:** Galina A. Eroshenko, Alina N. Balykova, Dmitriy N. Konanov, Levon A. Karapetyan, Lyubov M. Kukleva, Ekaterina A. Naryshkina, Nadezhda S. Chervyakova, Yaroslav M. Krasnov, Konstantin S. Shevchenko, Vladimir V. Kutyrev

**Affiliations:** 1 Federal State Institution of Science “Russian Anti-Plague Institute “Microbe”, Federal Service for Surveillance in the Sphere of Consumers Rights Protection and Human Welfare, Saratov 410005, Russiarusrapi@microbe.ru (V.V.K.); 2 Federal State Institution of Science “Research Institute for System Biology and Medicine”, Federal Service for Surveillance in the Sphere of Consumers Rights Protection and Human Welfare, Moscow 117246, Russia

**Keywords:** plague pathogen, strains, foci, Eastern Europe, Central Asia

## Abstract

Based on phylogenetic analysis of whole-genome sequencing of *Yersinia pestis* 2.MED1 strains of the medieval biovar, combined with epizootic, epidemiological, and climatic data over a 100-year period, we have reconstructed the most probable directions of distribution of plague in Eastern Europe and Central Asia (EECA) in the 20th and 21st centuries. The data suggest the important role of three great lakes—the Caspian, Aral, and Balkhash—in the circulation and preservation of *Y. pestis* 2.MED1 in EECA. Three main directions of *Y. pestis* 2.MED1 expansion have been identified: Caspian (Caspian Sea region foci, 1912–1945; Caucasus, 1953–1986), North Aral (Northern Aral Sea region foci, 1945–1959; Caspian Sea region foci, 1945–2015; Pre-Caucasus, 1999–2003; Karakum, 1949–1965) and Central Asian (Kyzylkum, 1924, 1983–2020; Balkhash foci, 1939–2020; Northern Aral Sea region foci, 1967–2020; Eastern Caspian Sea region foci, 1968–1985). Favorable climatic conditions in the Caspian Sea region, the Northern Aral Sea region, and the Balkhash region in the 20th and 21st centuries contributed to the rapid formation of stable natural plague foci and the long-term persistence of *Y. pestis* 2.MED1 strains of the medieval biovar, with their further introduction into other foci of EECA. Periodic introductions of the pathogen are one of the reasons for the plague re-emergence and activation of plague foci in the EECA region.

## 1. Introduction

There are 45 natural plague foci in Eastern Europe and Central Asia (EECA), located in Russia, Kazakhstan, Uzbekistan, Kyrgyzstan, Turkmenistan, Tajikistan, Azerbaijan, Armenia, and Georgia. Phylogeographic populations of the plague pathogen *Yersinia pestis* from these plague foci are diverse and belong to the biovars Antiqua and Mediavalis of the main subspecies *Y. pestis* ssp. *pestis* (phylogenetic lines 0.ANT, 4.ANT and 2.MED), and to the *caucasian* (0.PE2) and *central asian* (0.PE4) subspecies [1,2,3]. *Y. pestis* strains of the medieval biovar of the phylogenetic branch 2.MED1 are widespread in 32 of these foci (including 7 out of the 11 foci in Russia), located in the Caspian region, the Caucasus, and Central Asia [4]. These are highly virulent and epidemically hazardous strains. A comprehensive study of their properties and determination of the population structure and diversity of phylogeographic genotypes, routes of distribution and trends in modern evolution are important for improving the effectiveness of epidemiological monitoring and control of natural plague foci in the EECA countries.

The phylogenetic branch 2.MED1 is the most recent branch of evolution of the medieval biovar of *Y. pestis* (lineage 2.MED), which also includes the other evolutionary branches: 2.MED0, 2.MED2, 2.MED3, and 2.MED4. Strains 2.MED1 became widespread in the 20th century in EECA. Presumably, the beginning of this spread was the introduction of plague to the Russian port of Astrakhan in the Northern Caspian region in 1899 (or earlier in 1876 to the Vetlyanka railway station) from Iran. This led first to the large-scale distribution of plague in the Northern Caspian Sea region, later in the Western and Eastern Caspian Sea regions, the Caucasus, and Transcaucasia, and then in the Northern Aral Sea Region of Central Asia, where numerous plague foci emerged, manifesting epizootic and epidemic activity in the 20th century [1]. Some of them remain epizootically active and retain their epidemic significance in the 21st century [5].

The pathogen’s rooting in the Northern Caspian Sea region was facilitated by favorable climatic conditions: a high level of the Caspian Sea, abundant precipitation, soil moisture, and the availability of plant food for rodent carriers. However, by the late 1930s and early 1940s, the climate in the Northern Caspian Sea region began to change. The level of the Caspian Sea dropped dramatically, which had an adverse effect on the ecosystem of the Caspian Lowland and led to an increase in the average annual temperature, a decrease in the amount of grass feed supply for plague hosts, and a reduction in the number of rodents and their fleas. As a result, the epizootic activity of Northern Caspian foci decreased, and the incidence in this region declined. By the mid-20th century, the foci of the Caspian Sea entered an extended inter-epizootic period. In some foci, periods of rest lasted for several decades, and epizootic activity was only resumed in the middle of the second half of the 20th century. Based on phylogenetic analysis of whole-genome sequencing of *Y. pestis* strains, we have reconstructed the main patterns of circulation of 2.MED1 strains in the Northern Caspian region and the Caucasus in the 20th and early 21st centuries [6,7].

The aim of this study is to identify spatiotemporal patterns and possible causes of the distribution and persistence of *Y. pestis* 2.MED1 in EECA in the 20th and 21st centuries. Based on the results of a phylogenetic analysis of *Y. pestis* strains isolated over a period of more than 100 years, from 1912 to 2020, and epidemiological, epizootological, and climatic observations, we reconstructed a hypothetical distribution pattern for the 2.MED1 phylogenetic branch in EECA. The data assume the important role of three great lakes—the Caspian, Aral, and Balkhash—in the spread and preservation of *Y. pestis* 2.MED1 in the EECA countries.

## 2. Materials and Methods

### 2.1. Y. pestis Strains, Culture Conditions, Biochemical Analysis

Fifty *Y. pestis* strains of the medieval biovar isolated from carriers and vectors, and from humans in natural plague foci in EECA between 1912 and 2015, were used, 8 of which were sequenced in this study; the others were sequenced by us earlier. The strains were obtained from the working collection of the Laboratory of Molecular Microbiology of the Russian Anti-Plague Institute ‘Microbe’. The strains were isolated earlier from the little gopher *Spermophilus pygmaeus*, the midday gerbil *Meriones meridians*, the combed gerbil *M. tamariscinus*, the great gerbil *Rhombomys opimus*, and from other carriers and their fleas. Fourteen strains were also isolated from humans (Table S1). Cultivation of *Y. pestis* strains and analysis of their biochemical properties were carried out in accordance with conventional methods of laboratory diagnostics [8] and using a specialized API 20E kit manufactured by BioMerieux SA (Marcy-l’Étoile, France). The strains were grown at a temperature of 28 °C for 24–48 h in LB broth and on LB agar.

### 2.2. Whole-Genome Sequencing, SNPs’ Identification, Dendrogram Graphing

DNA of the strains for whole-genome sequencing was extracted using the PureLink Genomic DNA Mini Kit (Thermo Fisher Scientific, Waltham, MA, USA). Whole genome sequencing of the strains was carried out predominantly using the Ion GeneStudio S5 System (Thermo Fisher Scientific), according to the manufacturer’s guidelines. Ion Xpress™ Plus Fragment Library Kit and Ion Xpress™ Barcode Adapter 1-96 Kit were deployed for the primary preparation of samples. The Ion 510™ and Ion 520™ and Ion 530™ Chef Reagents were applied for automated template preparation. For each genome, raw short-read sequences were filtered and quality controlled using the Ion Torrent Suite v5.12.3 software package (https://github.com/iontorrent/TS (accessed on 6 May 2026)) and FastQC v0.11.9. (https://github.com/s-andrews/FastQC (accessed on 6 May 2026)). The de novo assembly of the obtained short sequences was performed using the Newbler gsAssembler 2.6 software package [9].

Whole-genome sequencing of *Y. pestis* strain 169 was performed on the DNBSEQ-G50RS (MGI, Shenzhen, China) platform using the MGIEasy Fast PCR-FREE FS Library Prep Set and DNBSEQ-G50 High-throughput Sequencing Set (FCL, PE150). Quality control and de novo assembly were performed using FastQC v 0.12.0. (https://github.com/s-andrews/FastQC (accessed on 6 May 2026)) and Unicycler v 0.5.0. (https://github.com/rrwick/Unicycler (accessed on 6 May 2026)). The genome assemblies and short reads of the *Yersinia pestis* strains sequenced in this study are available in NCBI GenBank Bioproject PRJNA1419569.

The average genome size was about 4.5 Mbp (Table S2). Core SNPs were identified through pairwise alignment of the genomes of the *Y. pestis* strains on the CO92 genome (NCBI GenBank accession number NC_003143) using the Snippy 4.6.0 program (https://github.com/tseemann/snippy (accessed on 6 May 2026)). The dendrogram was constructed using the Maximum Likelihood algorithm with the PhyML v3.1 program, which is built into SeaView v 5.0.5 [10], the GTR model, and 500 bootstrap replicates. The phylogenetic tree was visualized using the FigTree v1.4.4 software (http://tree.bio.ed.ac.uk/software/figtree/ (accessed on 6 May 2026)). The jModelTest2 program [11] was used to select the nucleotide substitution model. The GTR model was chosen based on the adjusted Akaike information criterion (AICc).

### 2.3. Determining the Time to Most Recent Common Ancestor (TMRCA)

All the genomes from the 2.MED1 branch were compared to the reference *Y. pestis* CO92 using Snippy 4.6.0. Only polymorphisms presented in at least two genomes were selected for further analysis. A phylogenetic tree was built using Fasttree 2.2 and analyzed using the Tempest software version 1.5.3 [12,13]. To estimate the TRMCA of the whole tree or individual clades, the tree was first rooted using ‘best-fitting root’ by optimizing the ‘heuristic residual mean squared’ function.

## 3. Results

**Figure 1:**
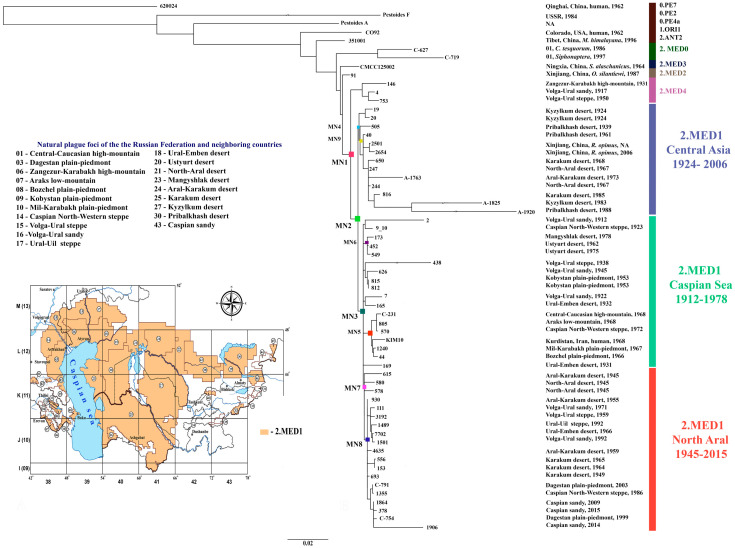
Dendrogram of relationships of the *Y. pestis* strains of the phylogenetic branch 2.MED1 isolated in the 20th–21st centuries in plague foci of Eastern Europe and Central Asia. Maximum Likelihood tree based on 1956 SNPs, GTR model with 500 bootstrap replicates. The numbers on the map indicate the number of natural plague foci in accordance with the classification adopted in the Russian Federation [3].

To construct a phylogenetic tree, whole-genome sequences of 50 *Y. pestis* strains of the medieval biovar isolated between 1912 and 2015 from plague foci in EECA in the Caspian Sea region, Caucasus, and Transcaucasia regions, and in the Aral Sea region, Balkhash, and other natural foci of Central Asia, were used. A dendrogram of phylogenetic relationships of the studied strains of the 2.MED1 branch was drawn using whole-genome analysis of 1956 identified core single polymorphisms (SNPs) (Figure 1). The identified core SNP mutations were both synonymous, not resulting in an amino acid substitution in the encoded protein, and non-synonymous, causing an amino acid substitution. Some of them were located in intergenic regions. The number and characteristics of individual SNPs for the phylogenetic nodes of the 2.MED1 branch on the dendrogram are presented in Table 1.

The dendrogram shows that a number of evolutionary branches of the medieval biovar diverge sequentially from the trunk of the phylogenetic lineage 2.MED: 2.MED0 (Central Caucasian high-mountain focus, Russia), 2.MED2 and 2.MED3 (foci of China), 2.MED4 (mainly foci of the Northwestern and Northern Caspian Sea regions, Russia, Kazakhstan, early 20th century) and 2.MED1 (foci of EECA) (Figure 1). Two late branches, 2.MED4 and 2.MED1, of the medieval biovar branch off in a common group at the phylogenetic node, designated by us as MN1 (Medieval Node). They are separated from the upstream branch 2.MED2 (strain 91, China) by seven SNPs located in the encoding sequences of the genes *acs*, *rimI*, *YPO_RS05220*, *ompF2*, *fadL*, *secD*, *YPO_RS21655*. Six of the seven nucleotide substitutions are not synonymous and cause a change in the amino acid sequence in the encoded product (Table 1).

Further, the 2.MED1 branch diverges from the general group with 2.MED4 in the phylogenetic node MN2 by eight specific SNPs located both in the coding sequences of the genes *YPO_RS05675* (s—synonymous), *cmoM* (s), *dinB* (ns—non-synonymous), *phnL* (s), *YPO_RS20180* (ns), and in the intergenic regions. The phylogenetic branch 2.MED1 itself includes strains isolated over a period of more than 100 years. The trunk of the 2.MED1 branch, in turn, is clearly divided into two branches, designated by us as Caspian and Central Asian. The first of them includes strains isolated mainly in the Caspian Sea region and the Caucasus, and the second in Central Asia.

The Caspian branch separates in the phylogenetic node MN3 by 7 marker SNPs located in the genes *sgrR* (s), *YPO_RS04025* (s), *YPO_RS09745* (s), *YPO_RS14495* (ns), *rfbH* (ns), *YPO_RS18110* (ns) and in the intergenic regions. The Caspian branch is initially represented by a basic polytomy consisting of strains from foci of the Caspian Sea region of the first half of the 20th century. The Caspian strains included in this polytomy were isolated in 1912–1945 in natural foci of the Northwestern (Caspian North-Western steppe focus) and Northern Caspian (Volga–Ural steppe, Volga–Ural sandy, and Ural–Emben desert foci) regions during the period of numerous outbreaks in the Caspian Lowland. The strains were isolated primarily from humans and are the etiological agents of those plague outbreaks. They were also isolated during that period from carriers and fleas, indicating their rooting in the natural ecosystems of the foci (Table S1). The strains of the first half of the 20th century exhibit genetic diversity and diverge from the trunk of the basic polytomy of the Caspian branch either as individual strains or in small clusters (Figure 1), which is shown in more detail in our other publication [6].

The Caspian branch also comprises strains from the foci of the Caucasus and Transcaucasia of the second half of the 20th century. This indicates its widespread distribution in the foci of the Caucasus and Transcaucasia at a time when such strains were already absent in the Northern and Northwestern Caspian region due to the onset of a long inter-epizootic period. The main cluster of Caucasian strains diverges from the basic polytomy of the Caspian branch at the MN5 node based on eight specific SNPs located both in the coding sequences of the *YPO_RS12430* (ns), *tatA* (s), YPO_RS20735 (ns), and *xylB* (ns) genes and in intergenic regions. This Caucasian cluster is represented by strains from the Mil-Karabakh and Bozchel autonomous foci of the Transcaucasian plain-piedmont focus (Azerbaijan, Georgia), and from the Araks low-mountain focus (Azerbaijan, Armenia). It also includes the KIM10 strain from Iran (Kurdistan, patient, 1968, GenBank). The structure of the 2.MED1 population in the Caucasian foci we described previously in more detail [7]. Caucasian 2.MED1 strains of the first half of the 20th century are unknown. The introduction of the Caspian 2.MED1 branch into the foci of the Caucasus and Transcaucasia could have occurred in various routes, primarily from the foci of the Northern and North-Western Caspian Sea regions, both by land and by sea. It is also possible that the Caspian branch strains reached Transcaucasia from the south, from the Iran–Kurdistan plague focus.

Another region where 2.MED1 strains of the Caspian branch were preserved in the second half of the 20th century was the territory of the Mangyshlak (Kazakhstan) and Ustyurt (Kazakhstan, Uzbekistan, and Turkmenistan) desert plague foci of the Eastern Caspian Sea region. In the dendrogram, strains from the Mangyshlak and Ustyurt foci of 1960–1970 are represented by a separate cluster, branching off from the trunk of the basic polytomy of the Caspian branch in the phylogenetic node MN6 and containing 4 specific SNPs in the coding sequences of the *YPO_RS07340* (ns) and *YPO_RS09555* (ns) genes and in the intergenic regions. Apparently, the cause for the preservation of strains of the Caspian branch here in the second half of the 20th century was favorable climatic conditions associated with the geographic location of these foci.

In the mid-20th century, a long inter-epizootic period began in the northern and northwestern Caspian foci, and plague outbreaks and epizootic manifestations ceased. We attribute this to climate warming, increasing aridity, and a dramatic drop in the Caspian Sea level, which led to changes in the ecosystem of the Caspian Lowland [14]. The dendrogram shows that at the beginning of that period, the basic polytomy of the Caspian branch gave rise to a new branch with two specific SNPs in the coding sequences of the *YPO_RS04355* (s) and *YPO_RS13470* (ns) genes (Figure 1, phylogenetic node MN7, Table 1). The new branch begins with the strains obtained from humans, carriers, and vectors in the foci of the Northern Aral Sea region in 1945. During this period, plague outbreaks began to occur in the Northern Aral Sea region for the first time. Phylogenetic analysis data indicate that the *Y. pestis* strains isolated in the foci of the Northern Aral Sea region (Kazakhstan) in 1945 were descendants of strains originating from the Northern Caspian Sea region. These strains took root in the natural ecosystem of the Northern Aral Sea region in the mid-20th century, due to favorable climatic conditions, and caused epizootics in rodents and human morbidity. During the 19th and 20th centuries, the Aral Sea level was consistently high [15,16], which created favorable conditions for the formation of natural plague foci. A distinctive feature of the Northern Aral Sea region strains of *Y. pestis* isolated in 1945 (strains 580, 615, and 578) was their independent divergence from the trunk of the emerging northern Aral branch and a large number of individual SNPs, despite the fact that the strains were isolated in September, October, and December 1945 in adjacent areas of the Kyzyl-Orda region.

The strains that took root in the Northern Aral Sea region (North Aral desert and Aral–Karakum desert foci) were preserved there in the mid-20th century during the inter-epizootic period that occurred at that time in the Northern Caspian Sea region. At the beginning of the second half of the 20th century, an increase in the Caspian Sea level led to the establishment of favorable climatic changes in the Caspian region, which resulted in the restoration of natural plague foci in the Caspian region after a long inter-epizootic period. Phylogenetic analysis data show that 2.MED1 strains of the second half of the 20th century from the Northern Caspian Sea region already belonged to the Northern Aral branch. They contained two genetic markers in their genomes: SNP mutations in the *YPO_RS04355* and *YPO_RS13470* genes, which are specific to this branch. This confirms the replacement of the *Y. pestis* 2.MED1 population of the Caspian branch by the Northern Aral branch in the second half of the 20th century in the Northern Caspian Sea region.

From the trunk of the Northern Aral branch that emerged around 1945, a new large polytomy (node MN8) with 3 SNPs in the genes *carA* (ns), *rssB* (s) and *YPO_RS13900* (ns) diverged, indicating the rapid distribution of the resulting population in various regions of the Caspian Sea region and Pre-Caucasus. The polytomy contains clusters of strains from the Northern and Northeastern Caspian regions (Volga–Ural sandy, Volga–Ural steppe and Ural–Uil sandy foci, Russia, Kazakhstan, 1959–1992), the Eastern Caspian region (Karakum desert focus, Turkmenistan, 1949–1965), the Northwestern Caspian region (Caspian North-Western steppe focus, Russia, 1986), the Western Caspian region (Caspian sandy focus, Russia, 2009–2015), and the Pre-Caucasus (Dagestan plain-piedmont focus, Russia, 1999–2009). The arrangement of the strains on the dendrogram reflects the consistent expansion of the 2.MED1 strains of the Northern Aral branch into the Caspian and Pre-Caucasus regions in the second half of the 20th century. Thus, the activation of these natural foci in the second half of the 20th century was caused by the introduction of 2.MED1 strains of the North Aral branch.

The 2.MED1 strains are clearly divided into two branches on the dendrogram—Caspian and Central Asian (Figure 1). The Caspian branch gave rise to the Northern Aral branch of 2.MED1 around 1945. The other, Central Asian branch is separated from the common trunk of 2.MED1 by only 1 SNP in the intergenic region—the phylogenetic node MN4 (Table 1). It follows from the dendrogram that the divergence and evolution of the Central Asian and Caspian branches occurred independently of each other. The distribution of strains of the Central Asian branch of 2.MED1 most likely followed a different route—from the south of Eurasia. This expansion led to the formation of natural foci of plague in the eastern part of the Central Asian region on the territory of the Turan Lowland, some of which still remain epizootically active.

The earliest known strains of 2.MED1 [17,18] of the Central Asian branch are associated with the plague outbreak in 1923 in the village of Ak-Kamysh (Uzbekistan) in the Kyzylkum desert focus. Subsequently, the 2.MED1 strains of the Central Asian branch reached the Balkhash region (Kazakhstan) and took root in the ecosystem of this region. Epizootics were first identified in the western part of the Balkhash desert focus in 1948 and in the Ili intermountain focus in 1930. In 1970, the level of Lake Balkhash began to rise [17,19,20], which was accompanied by the expansion of the Central Asian branch of 2.MED1.

The Central Asian branch is represented on the dendrogram by a large cluster of strains with diversification of smaller clusters within it according to a spatiotemporal principle. The *Y. pestis* 505 strain, isolated in the Balkhash focus in 1939, is phylogenetically related to the outbreak strains of 1923. A cluster including strain *Y. pestis* 40 (1961) from the Balkhash region and two strains, 2501 and 2654, from the Xinjiang Uyghur region of China of the early 21st century separates from it in the phylogenetic node MN9. Another subcluster of this cluster forms a small polytomy, including strains from the North Aral (1967), Karakum (1968), and Aral–Karakum (1973) desert foci. From the same polytomy, a cluster with strains from the Karakum (1985), Kyzylkum (1983), and Balkhash (1988) desert foci also diverges. The analysis of modern *Y. pestis* strains from NCBI GenBank, isolated mainly in 2017–2020 in the North Aral, Aral–Karakum, Kyzylkum desert foci, showed their belonging to the Central Asian 2.MED1 branch and close relationship with strains from the Balkhash desert focus (Figures S1 and S2).

The isolation of 2.MED1 strains of the Central Asian branch from 1924 to 2020 in the Balkhash, Kyzylkum, Karakum, North Aral, and Aral–Karakum foci demonstrates the widespread distribution of strains of this branch in Central Asia and the border foci of China. In some foci (the Karakum, North Aral, and Aral–Karakum desert foci), a change in 2.MED1 strains of the North Aral branch with strains of the Central Asian branch was observed, and the simultaneous circulation of both branches during the transition period of the 1960s–1970s. Apparently, the high ecological plasticity of *Y. pestis* strains of the medieval biovar of the 2.MED1 phylogenetic branch determined the possibility of their expansion in populations of a wide spectrum of rodent species found in the territories of the Caspian and Turan lowlands, the Balkhash–Alakol basin, and in the foothills, lowlands and highlands of the Pre-Caucasus, the Caucasus and even the Tien Shan mountains.

A temporal reconstruction based on single nucleotide polymorphism analysis of the core genome of the 2.MED1 strains used showed that the main clades arose in the first half of the 19th century (Figures S2 and S3). The calculated date of origin of the ancestral form of all used 2.MED1 strains is approximately 1820. The Central Asian branch 2.MED1 (1 specific SNP) arose around the same time, while the Caspian branch (7 specific SNPs) arose later in 1840. Both branches evolved independently (Figure 1, Figures S1 and S2) and their distribution areas practically do not overlap, with the exception of the northern Aral Sea region, which is adjacent to the territories of both branches.

## 4. Discussion

Climate has a significant impact on the parasitic system of natural plague foci and on its components, such as the causative agent, *Y. pestis*, rodent hosts, and arthropod vectors, and on the dynamics of their interaction in plague transmission cycles in nature. This issue has been the subject of numerous studies assessing the impact of ambient temperature, precipitation, soil moisture, and other climatic factors on the reproduction and interaction of hosts and vectors, and on the introduction of plague and the activation of natural foci, and global plague transmission in modern and historical periods [18,21,22,23,24,25,26]. However, understanding the impact of climate on plague still leaves more questions than answers. This article presents the data from our ongoing research to elucidate patterns of rapid plague distribution across vast expanses of the northern desert subzone of EECA in the Caspian and Turan lowlands and the Balkhash region in the 20th–21st centuries. The spread resulted in the formation of stable natural plague foci, where *Y. pestis* 2.MED1 circulates, accounting for 93.3% of all the plague foci in EECA. These strains are highly virulent and epidemiologically significant. At the very beginning of the 20th century, they caused numerous outbreaks of plague with high mortality in the Northern Caspian Sea region, and later caused outbreaks and cases of the disease in the Caucasus and Central Asian Republics (Table S3). The data of epidemiological, epizootological and climatic observations accumulated over a long period, combined with the capabilities of modern molecular–genetic methods for phylogenetic analysis of *Y. pestis* strains, provide a basis for identifying patterns of spatial and temporal distribution of *Y. pestis* strains, formation of natural foci and preservation of the pathogen in various landscape-geographic regions of Eurasia.

**Figure 2:**
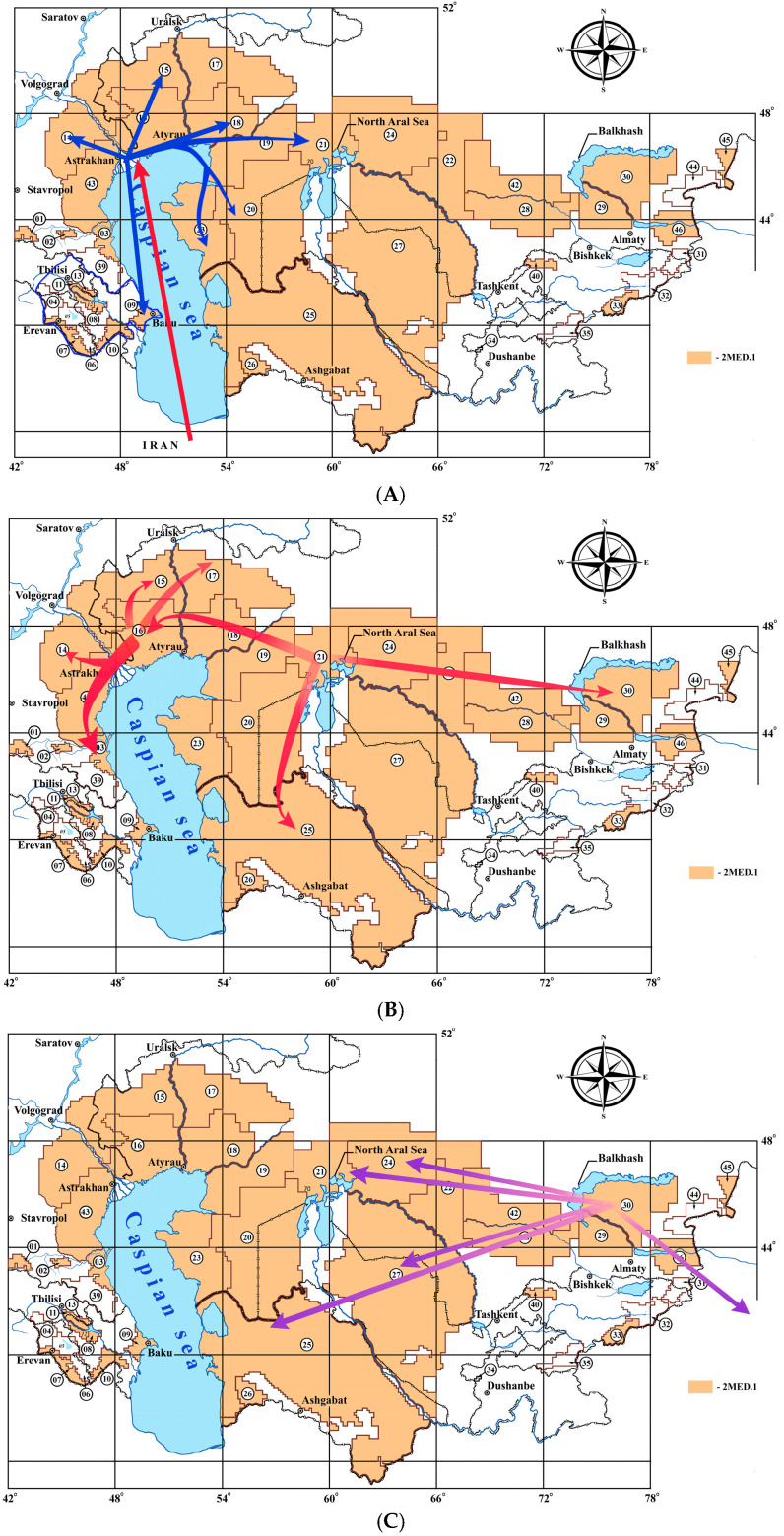
Hypothetical scheme of distribution of *Y. pestis* 2.MED1 strains in Eastern Europe and Central Asia: (**A**) Caspian Sea region, Caucasus and Transcaucasia. (**B**) Northern Aral Sea region. (**C**) Balkhash region. The numbers of the foci correspond to the designations in Figure 1.

The results presented in this paper are based on a study of more than 500 *Y. pestis* strains of the medieval biovar, the 50 most significant and key of which were used for the dendrogram construction based on their whole genome sequencing data (Figure 1). Also, the extended phylogenetic tree of 87 *Y. pestis* strains of the medieval biovar, including 16 strains (NCBI GenBank) of the 2.MED1 phylogenetic branch isolated mainly in 2017–2020, is shown in Figure S1. We established the presence of three phylogenetic branches—the Caspian, North Aral, and Central Asian—and suggest the existence of three main directions of *Y. pestis* 2.MED1 distribution in Eastern Europe and Central Asia. A temporal reconstruction showed that the Caspian branch arose approximately in 1840 (Figure S2) and was apparently introduced into the Northern Caspian region in 1899 to the town of Astrakhan or earlier in 1876 to the Vetlyanka station in the Astrakhan Governorate from the active foci of Iran. The introduced strains rooted in the Northern Caspian region (the Caspian Northwest Steppe, Volga–Ural steppe, and Volga–Ural sandy foci), which is confirmed by the isolation of strains in that period from carriers and vectors (small gopher, gerbils, voles and their fleas). The expansion of the introduced strains continued to other regions of the Caspian region: the Northeastern (Ural–Emba desert focus), Eastern (Ustyurt, Mangyshlak desert foci), Western (Caspian sandy focus), and to the Caucasus and Transcaucasia (Tersko-Sunzha low-mountain, Dagestan plain-piedmont, Central Caucasian high-mountain, Transcaucasian plain-piedmont, and Araks low-mountain foci) (Figure 2A).

The widespread distribution and persistence of 2.MED1 *Y. pestis* strains were facilitated by favorable climatic conditions in the Caspian region, including the high level of the Caspian Sea [14] and moderate average annual temperatures (Figure S4). This provided a good feed source for rodent carriers and also facilitated the breeding and abundance of fleas on rodents, which supported effective pathogen transmission in the rodent–flea–rodent triad. Against the backdrop of the prevailing climatic conditions, the 2.MED1 branch continued its eastward expansion in the northern desert subzone, reaching the Northern Aral Sea region, where it took root, forming the Northern Aral and Aral–Karakum desert foci. This is evidenced by the outbreaks of plague that first occurred in the Northern Aral Sea region in 1945 (Ak-Basty village in the Aral region of the Kzyl-Orda region—175 cases, 121 deaths), the source of which was field infections of the population or the slaughter of camels infected while grazing. The formation of the Northern Aral Sea foci occurred against the background of a consistently high level of the Aral Sea [15,16] in the 19th and 20th centuries (Figure S4). According to phylogenetic analysis, *Y. pestis* strains isolated from carriers, vectors, and sick people were descendants of the Northern Caspian strains but differed from them by two specific SNPs, which served as markers for the emerging Northern Aral branch (Figure 1, Table 1). The temporal reconstruction showed that the first strains of the North Aral branch arose around 1920 in the Northern Caspian region and then manifested themselves in 1945 with outbreaks in the Northern Aral Sea region (Figure 2A and Figure S2).

Currently, the water level of the Northern Aral Sea has risen due to its separation from the rest of the Aral Sea by a dam. The area of the Northern Aral Sea and its water reserves have increased significantly [16,27]. Climate warming has increased several times the flow of the Syr Darya River, which originates in the Tien Shan glaciers and feeds the Northern Aral Sea [28]. The rise in the water level of the Northern Aral Sea is consistent with the ongoing epizootic activity of the Northern Aral and Aral–Karakum foci in the 21st century [5,29].

In the middle of the 20th century, a long inter-epizootic period began in the foci of the Northern and North-Western Caspian regions, and outbreaks among people, and epizootics among rodents, ceased. Since 1930, the level of the Caspian Sea began to decline, fell by three meters by the middle 1940s, then, by 1977, it fell by another meter, but since 1978, it began to rise sharply. This factor largely affected the alternating phases of activity and inter-epizootic periods observed in the Caspian region in the 20th and early 21st centuries. The activation of plague foci in the Caspian region began in the second half of the 20th century and was caused by the expansion of strains from the Northern Aral Sea region. During this period, as the Caspian Sea level rose, climate changes occurred that were favorable for the restoration of natural plague foci after a long inter-epizootic period in the middle of the century. Strains 2.MED1 from the Northern Aral Sea region were introduced into the Northern Caspian region (and from there into the Western Caspian region and the Pre-Caucasus) after several decades of plague absence in these regions (Figure 2B). Strains from the Northern Aral Sea region were also introduced to the Eastern Caspian region, and from there across the Caspian Sea to the Transcaucasian plain-piedmont focus, which was accompanied by cases of plague and the rooting of new strains in the ecosystem of Transcaucasia along with the Northwestern, Northern and Eastern Caspian regions, and the Pre-Caucasus. Currently, the level of the Caspian Sea has reached its lowest point in the last 400 years, dropping to minus 29 m relative to the level of the World Ocean (https://tass.ru/obschestvo/245643 (accessed on 6 May 2026)). The foci of the Caspian region are now in deep depression.

The strains of another branch of 2.MED1, which we named Central Asian, arose approximately in 1820 and were introduced into Central Asia, apparently from South Asia. The initial distribution path of the Central Asian 2.MED1 branch is unknown, as there are no strains from the very early 20th century. The first plague outbreak (126 cases, 110 deaths) occurred in 1923 in the village of Ak-Kamysh in Karakalpakstan in Uzbekistan in the Kyzylkum desert focus. In 1929, another large outbreak of plague (151 cases, 126 deaths) occurred in the Dzharkent district of the Alma Ata region near the village of Kos-Agach [1]. The introduction of strains of the Central Asian branch led to the formation of the Balkhash desert and adjacent plague foci (Taukum and Muyunkum deserts) in the Balkhash–Alakol basin. Later, drifts from the Balkhash focus occurred repeatedly both to neighboring plague foci and over long distances to the Northern Aral Sea region and the Karakum desert focus, due to natural causes and in the process of economic activity with the involvement of automobile and railway transport (Figure 2C). From the Balkhash focus irradiation of the Central Asian branch of 2.MED1 occurred in North Aral (1967), Aral–Karakum (1973), Karakum (1968, 1985), Kyzylkum (1983) and other foci of Central Asia. The phylogenetic relationship of modern *Y. pestis* strains of the Central Asian 2.MED1 branch from the Balkhash, Kyzylkum, North Aral, and Aral–Karakum foci is shown in Figures S1 and S2. Since the 1970s and 1980s, precipitation levels in the Balkhash region have increased, and the lake’s area has expanded (Figure S2) [17,19,20]. Climate warming is now increasing the drainage of the largest Ili river, which flows into the Balkhash and originates in the Tien Shan Mountains. An increase in climate humidity has a positive effect on the ecosystem of the Balkhash region, on the food supply and the number of the main carrier, the great gerbil, and on the number of its fleas. The Balkhash Sea region plague foci were active in the 20th century and remain active in the current century [3,29].

## 5. Conclusions

Thus, favorable climatic conditions in the Caspian Sea region, the Northern Aral Sea region and the Balkhash region in the 20th and 21st centuries contributed to the formation of stable natural plague foci and the long-term persistence of *Y. pestis* 2.MED1 strains of the medieval biovar EECA. Currently, the foci of the Northern Aral Sea region (North Aral and Aral–Karakum desert foci) and Balkhash region (Balkhash, Ili, and Moyinkum desert foci) continue to show epizootic activity, which is associated with increased precipitation and high levels of the Northern Aral Sea and Balkhash. The data obtained indicate that local populations of *Y. pestis* may disappear under the influence of adverse climatic changes in the lowland, lowland-piedmont, and low-mountain natural foci located in arid landscapes of semi-deserts and deserts of the Caspian and Turanian lowlands. A new activation of epizootic processes occurs due to the introduction of the plague pathogen from other epizootically active territories and its subsequent rooting within the historical boundaries of natural plague foci or in new territories. In conclusion, it should be noted that the proposed model of the geographic distribution of *Y. pestis* in Eastern Europe and Central Asia in the 20th and 21st centuries is interpretive in nature and is based not solely on phylogenetic analysis but on a combination of epidemiological, ecological, and phylogenetic data. Identification of patterns of distribution and preservation of *Y. pestis* based on a comprehensive analysis of modern genetic studies of various phylogeographic populations of the pathogen and data of epidemiological, epizootological and climatic observations is important to make long-term forecasts for the activity of natural plague foci.

## Figures and Tables

**Table 1:** SNPs specific to the phylogenetic nodes of the 2.MED1 branch of *Y. pestis*.

Phylogenetic Node	Nucleotide Position in the CO92 Genome (NC_003143)	Nucleotide and Aminoacid Substitutions	Gene ID	Product
1	2	3	4	5
MN1	252309	C → T nsSNP ^1^ Gly128Ser	*acs*	Acetate-CoA ligase
	449523	C → A nsSNP Ala94Glu	*rimI*	Ribosomal protein S18-alanine N-acetyltransferase
	946150	C → A sSNP Ile364Ile	*YPO_RS05220*	Sugar AC transporter ATP-binding protein
	1356492	C → T nsSNP Arg58His	*ompF2*	Porin OmpF2
	3074598	C → T nsSNP Gly385Ser	*fadL*	Long-chain fatty acid transporter FadL
	3551475	G → A nsSNP Ala222Val	*secD*	Protein translocase subunit SecD
	4630765	G → T nsSNP Phe68Leu	*YPO_RS21655*	Amino acid ABC transporter permease
MN2	1040338	T → A sSNP Ala2158Ala	*YPO_RS05675*	C80 family cysteine peptidase
	1261149	C → T	Intergenic region (IR)	NA
	1586141	C → T sSNP Gly143Gly	*cmoM*	tRNA uridine 5-oxyacetic acid(34) methyltransferase CmoM
	2444694	C → A	MO IR	NA
	3596834	G → A nsSNP Ala325Val	*dinB*	DNA polymerase IV
	3805148	C → A	MO IR	NA
	3869550	C → A sSNP Gly156Gly	*phnL*	Phosphonate C-P lyase system protein PhnL
	4287044	G → T nsSNP Asp624Tyr	*YPO_RS20180*	Zinc/cadmium/mercury/lead-transporting ATPase
MN3	568345	A → T nsSNP Ser415Thr	*sgrR*	HTH-type transcriptional regulator SgrR
	664619	C → T nsSNP Trp149 ^2^	*YPO_RS04025*	Transporter
	1996390	C → T nsSNP Glu51Lys	*YPO_RS09745*	SPAS domain S-box protein
	2269099	G → T	MO IR	NA ^3^
	3030163	C → T nsSNP Gln140 ^2^	*YPO_RS14495*	DUF979 domain-containing protein
	3470222	G → A nsSNP Ser85Phe	*rfbH*	Lipopolysaccharide biosynthesis protein RfbH
	3833893	C → A nsSNP Arg97Leu	*YPO_RS18110*	GMP reductase
MN4	3399782	T → C	MO IR	NA
MN5	573525	A → G	MO IR	NA
	2565275	G → A nsSNP Ala105Val	*YPO_RS12430*	Substrate-binding domain-containing protein
	2918692	G → A sSNP Leu19Leu	*tatA*	Sec-independent protein translocase subunit TatA
	3448819	G → C	MO IR	NA
	4067118	C → T	MO IR	NA
	4262988	T → A	MO IR	NA
	4403326	T → C nsSNP Asn500Asp	*YPO_RS20735*	Type I secretion system permease/ATPase
	4556160	C → A nsSNP Leu292Ile	*xylB*	Xylulokinase
MN6	472490	G → C	MO IR	NA
	1424616	G → T nsSNP Gln96His	*YPO_RS07340*	Bcr/CflA family multidrug efflux MFS transporter
	1961220	A → T nsSNP Asn2Lys	*YPO_RS09555*	L-cystine transporter
	4589546	G → A	MO IR	NA
MN7	739446	C → T sSNP Phe35Phe	*YPO_RS04355*	Hypothetical protein
	2803151	G → A nsSNP Arg338Cys	*YPO_RS13470*	BCCT family transporter
MN8	512182	G → T nsSNP Ala26Ser	*carA*	Glutamine-hydrolyzing carbamoyl-phosphate synthase small subunit
	2445173	C → T sSNP Leu41Leu	*rssB*	Two-component system response regulator RssB
	2911550	C → T nsSNP Arg231Cys	*YPO_RS13900*	ABC transporter transmembrane domain-containing protein
MN9	935289	C → A nsSNP Ser394 ^2^	*YPO_RS05180*	Maltoporin
	4166505	C → T sSNP Ala102Ala	*metH*	Methionine synthase
	4343116	C → T	MO IR	NA

^1^ ns—non-synonymous substitution; s—synonymous substitution; the number indicates the position of the amino acid in the protein sequence. ^2^ stop codon. ^3^ NA—not available.

## Data Availability

The genome assemblies and short reads of *Yersinia pestis* strains sequenced in this study are available in NCBI GenBank Bioproject PRJNA1419569.

## Supplementary Materials

The following supporting information can be downloaded at: https://www.mdpi.com/article/10.3390/pathogens15060568/s1. Table S1: *Y. pestis* strains used in this work. (DOCX); Table S2: Accession numbers and basic statistics of *Y. pestis* genomes (XLSX); Table S3. The first epidemiological and epizootological manifestations of plague in the foci of Eastern Europe and Central Asia. (DOCX); Figure S1: Extended phylogenetic tree of 87 *Y. pestis* strains of the medieval biovar, including 16 strains (NCBI GenBank) of the 2.MED1 phylogenetic branch isolated in the 21st century. Maximum Likelihood algorithm. GTR model with 500 bootstrap replicates (JPG); Figure S2: SNP-based phylogenetic tree of the used 2.MED1 strains, isolated in 1912–2020 in plague foci of Eastern Europe and Central Asia. Only SNPs presented in more than one strain were used. Molecular dating of the reconstructed ancestors was performed using TempEst [14]. Figure S3: SNPs heatmap of the 2.MED1 strains isolated in 1912–2020 in plague foci of Eastern Europe and Central Asia. Figure S4: Levels of the Caspian, Aral and Balkhash lakes in the 20th–21st centuries. The abscissa axis shows the years of observations, and the ordinate axis shows the fluctuations in water level relative to the world level. A. Caspian Lake. B. Aral Lake. C. Lake Balkhash (JPG).
