# Supplementary material for: The Role of the Caspian, Aral and Balkhash Lakes in the Spread and Preservation of Yersinia pestis in Eastern Europe and Central Asia in the 20th and 21st Centuries
Source: Pathogens. 2026 May 25;15(6):568. doi: 10.3390/pathogens15060568 (PMC13304539; doi:10.3390/pathogens15060568)
Supplement: Supplementary file 1 [file pathogens-15-00568-s001.zip › Table S1.pdf]

№	Strain	Plague focus, geographical region of isolation	Source, year of isolation	Phylogenetic branch, access number NCBI GenBank
1	C-627	Central-Caucasian high-mountain, Kabardino-Balkarian Republic, RF	1986, *Citellophilus tesquorum*	2.MED0 MBSI00000000
2	C-754	Dagestan plain-piedmont, Republic of Dagestan, RF	1999, *Neopsylla setosa*	2.MED1 JABUII000000000
3	C-791	Dagestan plain-piedmont, Republic of Dagestan, RF	2003, *Spermophilus pygmaeus*	2.MED1 LQAU00000000
4	146	Zangezur-Karabakh high-mountain, Azerbaijan , Nagorno-Karabakh	1931, human	2.MED4 JABTYS000000000
5	805	Araks low-mountain, Nakhichevan Autonomous Republic	1968, *Meriones vinogradovi*	2.MED1 JALMFF000000000
6	44	Boschel plain-piedmont, Azerbaijan	1966, *Ceratophyllus laeviceps*	2.MED1 LZNF00000000
7	812	Kobystan plain-piedmont, Azerbaijan	1953, *Stenoponia insperata*	2.MED1 JALMFI000000000
8	815	Kobystan plain-piedmont, Azerbaijan	1953, *Ctenophthalmus secundus*	2.MED1 JALMFJ000000000
9	1240	Mil-Karabach plain-piedmont, Azerbaijan	1967, *Meriones erythrourus*	2.MED1 LZNI00000000
10	9_10	Caspian North-Western steppe, Rostov region, RF	1923, human	2.MED1 WUCK00000000
11	570	Caspian North-Western steppe, Ketchenerovsky region, Republic of Kalmykia, RF	1972, Siphonaptera	2.MED1 JALMFO000000000
12	M-1355	Caspian North-Western steppe, Republic of Kalmykia, RF	1986, *Citellophilus tesquorum*	2.MED1 JABUIK000000000
13	438	Volga-Ural steppe, Astrakhan region, RF	1938, *Microtus arvalis*	2.MED1 JABUHY000000000
14	3192	Volga-Ural sSteppe, Atyrau Region, Kazakhstan	1959, *Spermophilus pygmaeus*	2.MED1 JABUIF000000000
15	753	Volga-Ural steppe; West Kazakhstan Region, Kazakhstan	1950, *Spermophilus pygmaeus*	2.MED4 JABUIB000000000
16	2	Volgo-Ural sandy, West Kazakhstan	1912, human	2.MED1 JABTYO000000000
17	4	Volgo-Ural sandy, West Kazakhstan	1917, *Camelus*	2.MED4 JABTYP000000000
18	7	Volgo-Ural sandy, West Kazakhstan	1922, human	2.MED1 JAAIKX000000000
19	626	Volga-Ural sandy, Atyrau Region, Kazakhstan	1945, human	2.MED1 JABUIA000000000

20	111	Volgo-Ural sandy, Kazakhstan	1971, *Meriones tamariscinus*	2.MED1 WUCM00000000
21	M-1501	Volgo-Ural sandy, Kazakhstan	1992, *Meriones meridianus*	2.MED1 JABUIN000000000
22	M-1489	Ural-Uil steppe, Kazakhstan	1992, *Rhombomys opimus*	2.MED1 JABUIM000000000
23	169	Ural-Emben desert, Atyrau Region, Kazakhstan	1931, human	2.MED1 JBUXKM000000000
24	165	Ural-Emben desert, Atyrau Region, Kazakhstan	1932, human	2.MED1 JABTYT000000000
25	7702	Ural-Emben desert, Atyrau Region, Kazakhstan	1966, *Spermophilus pygmaeus*	2.MED1 JABUIH000000000
26	452	Ustyurt Desert; Mangystau Region, Kazakhstan	1962, *Rhombomys opimus*	2.MED1 JALMFQ000000000
27	549	Ustyurt Desert; Balkan Province, Turkmenistan	1975, *Rhombomys opimus*	2.MED1 JALMFR000000000
28	578	North-Aral desert, Kyzylorda region, Kazakhstan	1945, human	2.MED1 JAAIKW000000000
29	580	North-Aral desert, Kyzylorda region, Kazakhstan	1945, human	2.MED1 JABUHZ000000000
30	244	North-Aral desert, Aralsk, Kazakhstan	1967, human	2.MED1 LZND00000000
31	247	North-Aral desert, Aralsk, Kazakhstan	1967, camel	2.MED1 JBUXKI000000000
32	173	Mangyshlak desert, Kazakhstan	1978, *Rhombomys opimus*	2.MED1 LQAZ00000000
33	615	Aral-Karakum desert; Kyzylorda Region, Kazakhstan	1945, *Rhombomys opimus*	2.MED1 WUCL00000000
34	930	Aral-Karakum desert, Kazakhstan	1955, *Meriones meridianus*	2.MED1 JABUIJ000000000
35	4635	Aral-Karakum desert, near Aralsk, Kazakhstan	1959, *Haemaphysalis*	2.MED1 JABUIG000000000
36	A-1763	Aral-Karakum desert, Kazakhstan	1973, *Rhombomys opimus.*	2.MED1 LQAW00000000
37	693	Karakum desert; Balkan Province, Turkmenistan	1949, human	2.MED1 JABWHH000000000
38	153	Karakum desert, Turkmenistan	1964, *Meriones erythrourus*	2.MED1 JABWHI000000000
39	M-556	Karakum desert; Dashoguz Province, Turkmenistan	1965, human	2.MED1 JBUXKL000000000
40	650	Karakum desert, Uzbekistan	1968, *Ceratophyllus laeviceps*	2.MED1 JBUXKK000000000
41	KM-816	Karakum desert; Balkan Province, Turkmenistan	1985, *Rhombomys opimus*	2.MED1 LPXU00000000
42	19	Kyzylkum Desert; Ak Kamysh, Turkmenistan	1924, human	2.MED1 JBUXKF000000000

43	20	Kyzylkum Desert; Ak Kamysh, Turkmenistan	1924, human	2.MED1 JBUXKG000000000
44	A-1825	Kyzylkum Desert, Uzbekistan	1983, *Rhombomys opimus*	2.MED1 LYCM00000000
45	505	Balkhash desert, Almaty Region, Kazakhstan	1939, *Rhombomys opimus*	2.MED1 JBUXKJ000000000
46	40	Balkhash desert, Kyrgyz Republic	1961, *Rhombomys opimus*	2.MED1 JBUXKH000000000
47	A-1920	Balkhash desert, South Balkhash region, Kazakhstan	1988, Siphonaptera	2.MED1 LYCO00000000
48	M-1864	Caspian sandy; Lagansky district, Republic of Kalmykia, RF	2009, *Meriones meridianus*	2.MED1 LOHR00000000
49	378	Caspian sandy, Chernozemelsky district, Republic of Kalmykia, RF	2015, *Nosopsylla laeviceps*	2.MED1 JABUHX000000000
50	1906	Прикаспийский песчаный; Лаганский р-он, Респ. Калмыкия, РФ	2014, *Meriones meridianus*	2.MED1 LYOM00000000
**NCBI GenBank**				
1	C-719	Central Caucasian mountain, Kabardino-Balkarian Republic, RF	1997, Siphonaptera	2.MED0 VWRY00000000
2	C-231	Bozchel plain-piedmont Geranbol district, Azerbaijan	1968, *Mesopsylla apscheronica*	2.MED0 VEZW00000000
3	KIM10	Kurdistan, Iran	1968, human	2.MED1 NC_004088.1
4	2501	Xinjiang Uyghur Autonomous Region, China	*Rhombomys opimus*	2.MED1 AKVQ00000000
5	2654	Xinjiang Uyghur Autonomous Region, China	2006, *Rhombomys opimus*	2.MED1 ADPB00000000
6	620024	Qinghai, China	1962, human	0.PE7 ADPM00000000
7	Pestoides A	Former Soviet Union	Not available	0.PE4 ACNT00000000
8	Pestoides F	Former Soviet Union	<1984	0.PE2 NC_009381.1
9	351001	Tibet, China	1996, *Marmota himalayana*	2.ANT2 ADPF00000000
10	CO92	Colorado, USA	1992, human	1.ORI1 NC_003143.1
11	CMCC125002	Ningxia, China	1964, *Spermophilus alaschanicus*	2.MED3 ADQN00000000
12	91	Xinjiang Uyghur Autonomous Region, China	1987, *Oropsylla silantiewi*	2.MED2 ADPU00000000
