# Supplementary material for: The Role of the Caspian, Aral and Balkhash Lakes in the Spread and Preservation of Yersinia pestis in Eastern Europe and Central Asia in the 20th and 21st Centuries
Source: Pathogens. 2026 May 25;15(6):568. doi: 10.3390/pathogens15060568 (PMC13304539; doi:10.3390/pathogens15060568)
Supplement: Supplementary file 1 [file pathogens-15-00568-s001.zip › Table S2.pdf]

Table S2. Accession numbers and basic statistics of *Y. pestis* genomes

Strain	Platform	Read type	Read avg length	Total reads	Toltal assembly length	No. of contigs	No. of contigs >1 kb	N50	Avg depth	SRA accession no.
19	Ion GeneStudio S5 System	single-end	246	1304304	4475869	214	163	47965	71,69	SRR37144234
20	Ion GeneStudio S5 System	single-end	244	1791850	4463030	186	160	48943	97,96	SRR37144233
40	Ion GeneStudio S5 System	single-end	286	595345	4573181	217	166	48450	37,23	SRR37144235
247	Ion GeneStudio S5 System	single-end	264	1567599	4575440	190	150	49371	90,45	SRR37144236
505	Ion GeneStudio S5 System	single-end	257	740169	4453882	210	163	44427	42,7	SRR37144237
650	Ion GeneStudio S5 System	single-end	267	1412729	4549070	188	159	48673	82,92	SRR37144238
556	Ion GeneStudio S5 System	single-end	213	765313	4543822	197	184	41302	35,88	SRR37144239
169	MGI (DNBSEQ-G50RS)	paired-end	150	2249744	4580570	193	145	51275	73,67	SRR37144240
