# Supplementary material for: The Role of the Caspian, Aral and Balkhash Lakes in the Spread and Preservation of Yersinia pestis in Eastern Europe and Central Asia in the 20th and 21st Centuries
Source: Pathogens. 2026 May 25;15(6):568. doi: 10.3390/pathogens15060568 (PMC13304539; doi:10.3390/pathogens15060568)
Supplement: Supplementary file 1 [file pathogens-15-00568-s001.zip › Table S3.pdf]

Foci/number	Country/region/area/ outbreak	Epizootics
Dagestan plain–piedmont (03)	Russia, Dagestan, Babayurt district — 1951	Kumyk and Prisulak lowlands — 1951–1952
Transcaucasian high-mountain (04–06)	Armenia, Spitak district — 1958	Gyumri mesofocus — 1958
	Armenia, Leninakan district — 1969, 1975	Zangezur–Karabakh mesofocus — 1962
		Prisevan mesofocus — 1962
Transcaucasian plain-piedmont (08–13)	Azerbaijan, Baku district — 1914	Apsheron — 1914, 1949, 1952
	Nagorno-Karabakh — 1929	
Caspian Northwestern steppe (14)	Russia, Astrakhan Region — 1912	1913–1948
	Russia, Volgograd Region — 1913	
	Russia, Republic of Kalmykia — 1929–1931	
	Russia, Rostov Region, Zavetinsky district — 1922–1925	
	Russia, Stavropol Territory — 1932	
Volga–Ural steppe (15)	Russia, Astrakhan Region — 1876–1879, 1900	1912–1950
	Russia, Volgograd Region — 1899	
	Kazakhstan, Atyrau Region — 1925	
	Kazakhstan, West Kazakhstan Region, Kaztalov district — 1925	
Volga–Ural sandy (16)	Russia, Astrakhan Region — 1899, 1905, 1906	1922–1923
	Kazakhstan, Atyrau Region, Novobogatinsky district — 1899, 1903, 1907	
	Kazakhstan, West Kazakhstan Region — 1909, 1910	
Ural–Uil steppe (17)	Kazakhstan, Aktobe Region, Chelkar district — 1929	1913–1915
	Kazakhstan, West Kazakhstan Region, Taipak district — 1913	
	Kazakhstan, Atyrau Region — 1915	
Caspian sandy (43)	Russia, Astrakhan Region — 1923	1923–1925
	Russia, Republic of Kalmykia — 1924	
Ural–Embinsky desert (18)	Kazakhstan, Atyrau Region, Makat district — 1955	1951
Ustyurt desert (20)	Kazakhstan, Atyrau Region — 1975	1958
North Aral desert (21)	Kazakhstan, Kyzylorda Region, Aral district — 1945, 1955	1945–1950
Mangyshlak desert (23)	Kazakhstan, Atyrau Region — 1926, 1927	1926–1927
Aral-Karakum desert (24)	Kazakhstan, Kyzylorda Region — 1966, 1969	1947
Karakum desert (25)	Turkmenistan, Mary Region — 1912	1949–1950
	Turkmenistan, Krasnovodsk Region — 1953	

Kyzylkum desert (27)	Uzbekistan, Karakalpakstan — 1923, 1947	1923, 1947–1950
Pre-Balkhash desert (30)	Kazakhstan, Almaty Region — 1929, 1947	1939, 1947–1951
Talas high-mountain (40)	No epidemic manifestations of plague recorded in natural focus	Talas Range — 1977

Table S3. The first epidemiological and epizootological manifestations of plague in the foci of Eastern Europe and Central Asia
