# Supplementary figures and images for: The Role of the Caspian, Aral and Balkhash Lakes in the Spread and Preservation of Yersinia pestis in Eastern Europe and Central Asia in the 20th and 21st Centuries
Source: Pathogens. 2026 May 25;15(6):568. doi: 10.3390/pathogens15060568 (PMC13304539; doi:10.3390/pathogens15060568)

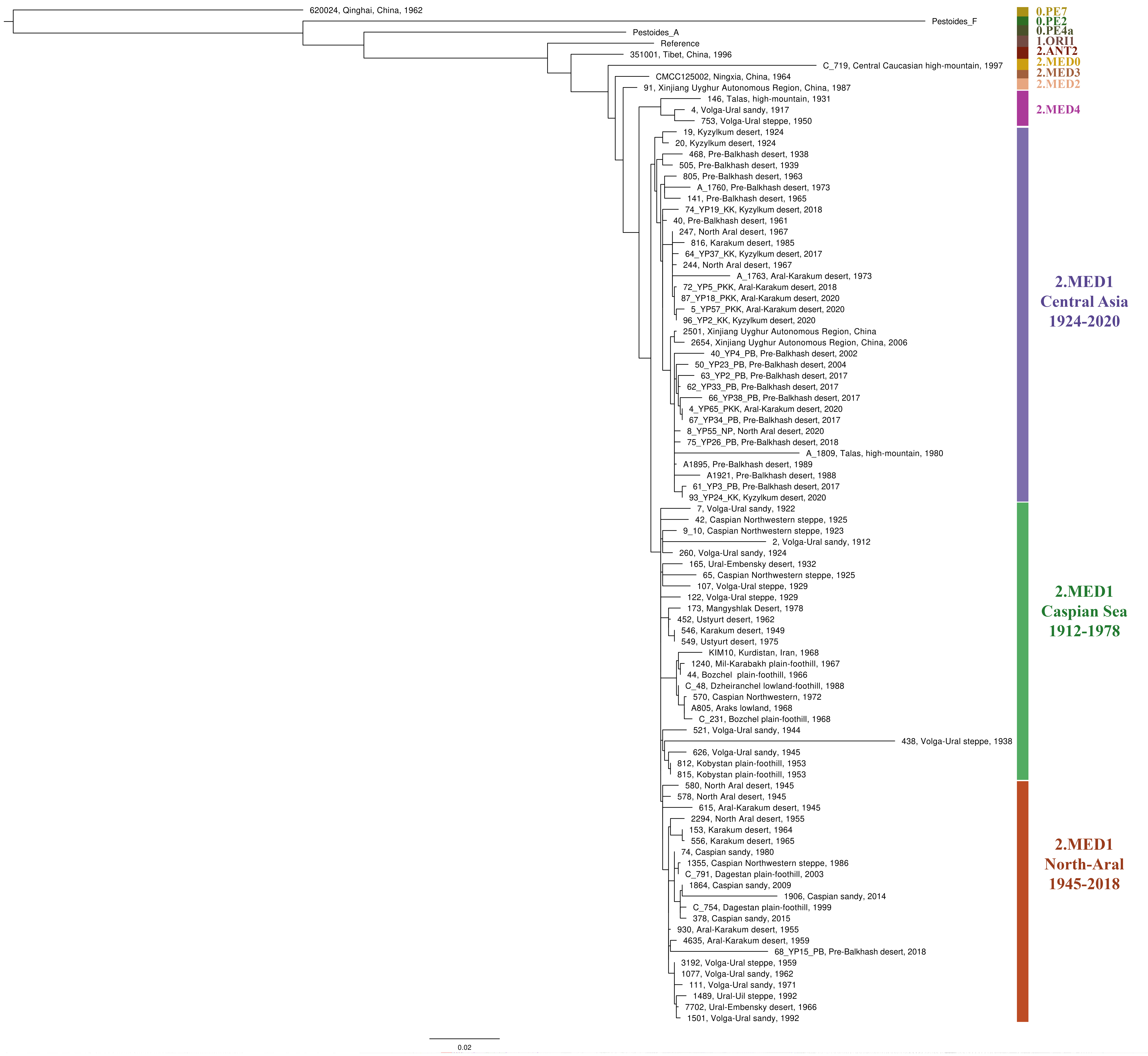

Supplement: Supplementary file 1 [file pathogens-15-00568-s001.zip › Figure S1.jpg]

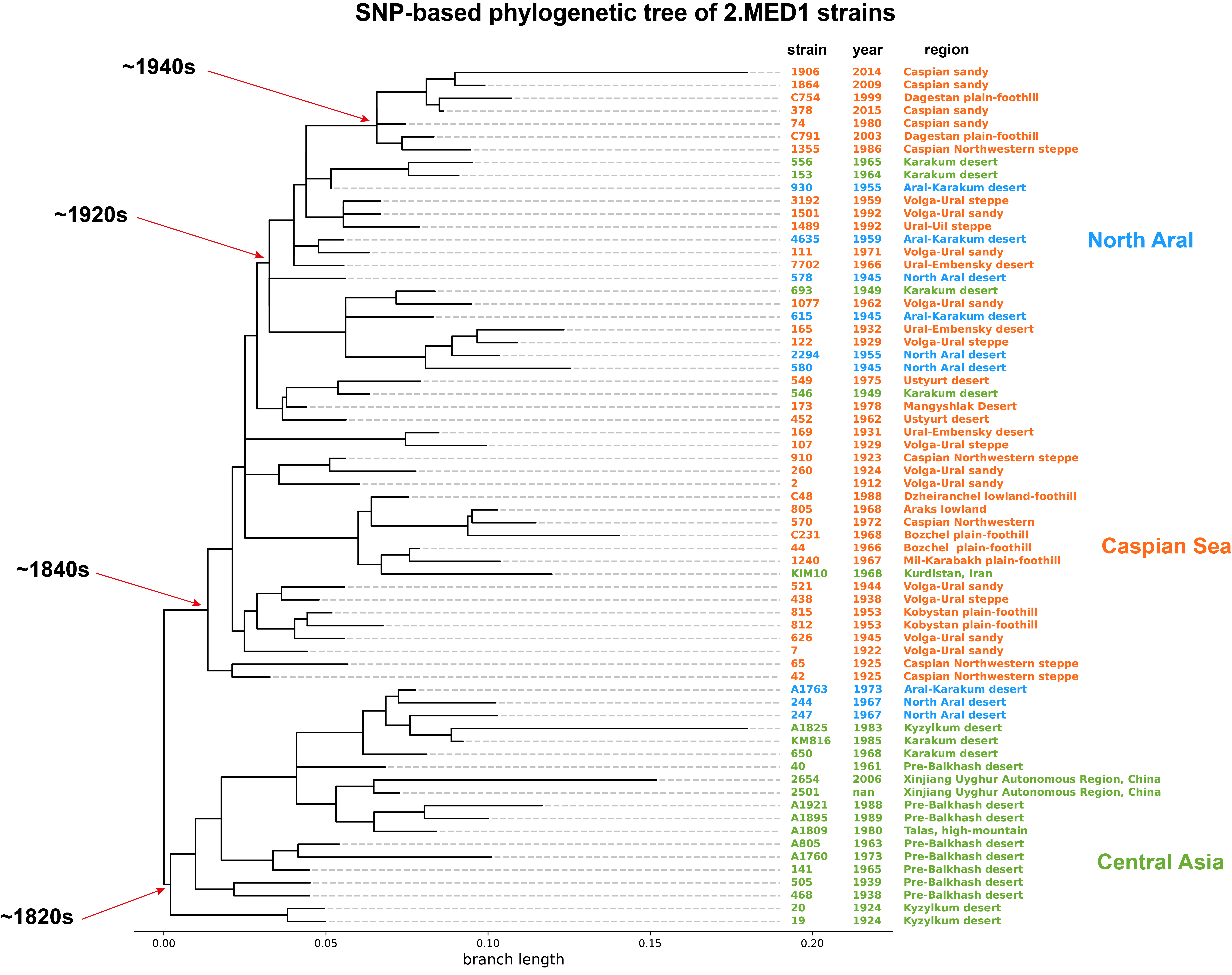

Supplement: Supplementary file 1 [file pathogens-15-00568-s001.zip › Figure S2.jpg]

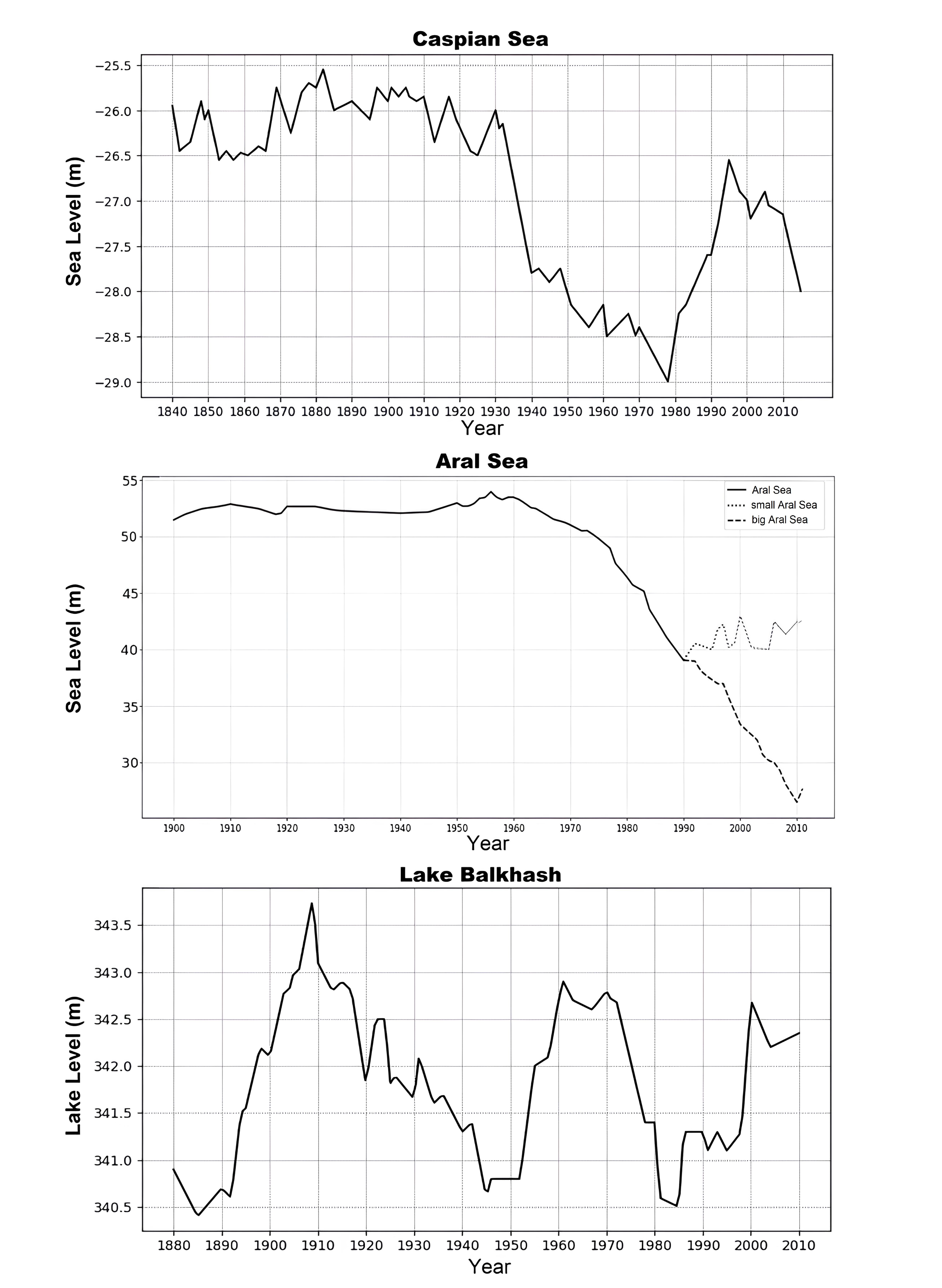

Supplement: Supplementary file 1 [file pathogens-15-00568-s001.zip › Figure S4.jpg]
